# Author Correction: Inferring ecosystem networks as information flows

**DOI:** 10.1038/s41598-021-94762-9

**Published:** 2021-07-30

**Authors:** Jie Li, Matteo Convertino

**Affiliations:** 1 Nexus Group, Faculty and Graduate School of Information Science and Technology, Hokkaido University, Sapporo, Japan; 2 GI-CORE Global Station for Big Data and Cybersecurity, Hokkaido University, Sapporo, Japan; 3 Graduate School of Information Science and Technology, Hokkaido University, 9 Chome, Kita 14, Nishi 9, Kita-ku, room 11-11, Sapporo, Hokkaido 060-0814 Japan; 4 Institute of Environment and Ecology, Tsinghua Shenzhen International Graduate School, Tsinghua University, Shenzhen, China

Correction to: *Scientific Reports* https://doi.org/10.1038/s41598-021-86476-9, published online 29 March 2021

In the original version of this Article, the Affiliations given for the author Matteo Convertino were incomplete.

The correct affiliations are listed below:

Affiliation 1 Nexus Group, Faculty and Graduate School of Information Science and Technology, Hokkaido University, Sapporo, Japan.

Affiliation 2 GI-CORE Global Station for Big Data and Cybersecurity, Hokkaido University, Sapporo, Japan.

Affiliation 3 Graduate School of Information Science and Technology, Hokkaido University, 9 Chome, Kita 14, Nishi 9, Kita-ku, room 11-11, Sapporo, Hokkaido 060-0814, Japan.

Affiliation 4 Institute of Environment and Ecology, Tsinghua Shenzhen International Graduate School, Tsinghua University, Shenzhen, China.

The original Article has been corrected.

